# A re-examination of the West European species of *Boreonectes* Angus, 2010, with particular reference to *B. multilineatus* (Falkenström, 1922) (Coleoptera, Dytiscidae)

**DOI:** 10.3897/compcytogen.v15.i1.60188

**Published:** 2021-01-14

**Authors:** Robert B. Angus

**Affiliations:** 1 Department of Life Sciences (Insects), the Natural History Museum, Cromwell Road, London SW7 5BD, UK the Natural History Museum London United Kingdom

**Keywords:** *
Boreonectes
*, *B. multilineatus*, chromosomes, Coleoptera, Distributions, Dytiscidae, Pyrenees, Pleistocene fossils

## Abstract

The West European species of *Boreonectes* Angus, 2010 are reviewed. *B. multilineatus* (Falkenström, 1922) is shown to be widely distributed in the Pyrenees, where it is the only species known to occur. The chromosomes of all five west European species are found to have, in addition their different numbers of chromosomes, differences in the number and locations of secondary constrictions, and in some cases, the number of chromosomes with clear centromeric C-bands. The level of differences between the chromosomes of the species is in stark contrast with the very slight genetic (DNA) differences between them and this suggests that chromosome differentiation may have been a driver of speciation. Two of the species, *B. griseostriatus* (De Geer, 1774) and *B. multilineatus*, have distributions extending northwards as far as Arctic Scandinavia. It is pointed out that, while these northern areas now constitute the major portions of their ranges, they must be of fairly recent origins as most of the area would have been covered by ice sheets and therefore not habitable during the glacial maximum of the Last Glaciation. This contrasts with the situation in the area of the Central European mountains where fossil faunas, including *Boreonectes*, are known. *B. griseostriatus*, identifiable to species by its parameres, was present in the Woolly Rhinoceros site at Starunia in the Western Ukraine, and this fauna is discussed as well as an English fauna of similar age.

*For Ignacio Ribera, 1963–2020. Taken from us by the Corona virus when he still had so much research to enjoy and to share with the rest of us*.

## Introduction

*Boreonectes* Angus, 2010 is a genus of small diving beetles (Dytiscidae) typically found in mountain lakes and in barren pools at lower altitudes in the north. The Palaearctic species were for many years regarded as all belonging to a single species, *B. griseostriatus* (De Geer, 1774) (see Zaitzev 1953 for discussion), though their generic placement was not stable until Angus (2010b) erected the genus *Boreonectes*. Fery and Ribera (2018), in their analysis of the genera of subtribe Deronectina, further stabilised *Boreonectes* by restricting it to the *B. griseostriatus* group plus one other species. However, chromosomal studies started by R. B. Angus in the 1980s began to show a number of different karyotypes. The results of these studies were published by L. A. Dutton and R. B. Angus (2007) and revealed the existence of seven distinct species, five of them new, in Europe. Details of the Palaearctic species are given in Table 1.

**Table 1.** The Palaearctic species of *Boreonectes* Angus, 2010.

No	**Species**	**Distribution**
1	* Boreonectes griseostriatus* (De Geer, 1774)	Fennoscandia, Alps (northern)
	= *B. maritimus* (Helliesen, 1890)	
	= * B. griseostriatus * var. *nigrescens* Favre, 1890	
1a	* B. griseostriatus griseostriatus* (De Geer, 1774)	
1b	* B. griseostriatus strandi* (Brink, 1943)	Arctic coast of Fennoscandia and Kola
2	* Boreonectes multilineatus* (Falkenström, 1922)	Pyrenees, Inland Scandinavia, British Isles, Faroes
3	* Boreonectes emmerichi* (Falkenström, 1936)	Tibetan Plateau
4	* Boreonectes macedonicus* (Guéorguiev, 1959)	North Macedonia, Crete
	= *B. creticus* (Dutton et Angus, 2007)	
5	* Boreonectes alpestris* (Dutton et Angus, 2007)	Alps (southern)
6	* Boreonectes ibericus* (Dutton et Angus, 2007)	Mountains of Iberia, Maritime Alps, Corsica, Middle Atlas Mts
7	* Boreonectes inexpectatus* (Dutton et Angus, 2007)	France, Hautes Alpes, Lac du Lauzet inférieur
8	* Boreonectes riberae* (Dutton et Angus, 2007)	Bulgaria, Turkey (Anatolia)
9*	* Boreonectes piochardi* (Régimbart, 1878)	Israel/Lebanon, Mt Hermon
10*	* Boreonectes palaestinus* (Baudi di Selve, 1894)	Palestine, Syria

*Footnote. These two names almost certainly refer to the same species and the type of *B. piochardi* is a *Boreonectes*. From their distributions it seems possible that they are the same species as *B. riberae*, over which they have priority. Chromosome preparations from living material would be needed to resolve this.

*B. multilineatus* (Falkenström, 1922) is among these chromosomally validated species, and was taken to refer to paler, more distinctly striped material from inland montane areas in Fennoscandia, with *B. griseostriatus* occurring in coastal rocky localities (Nilsson and Holmen 1995). Nilsson and Holmen added that while *B. griseostriatus* was not known outside coastal Fennoscandia, *B. multilineatus* was in all probability the species recorded across northern Eurasia as far east as Kamchatka. However, serious doubt was cast on this view by the discovery by Angus (2008) of pale, strongly lined *B. griseostriatus*, closely resembling *B. multilineatus*, near Sevettijärvi in Finnish Lapland. Not only that, but *B. griseostriatus* is now known to be widely distributed in the northern part of the Alps, from the Col du Petit St Bernard in the west to Bavaria in the east (Angus 2010a, b) and Franck Bameul’s discovery of *B. multilineatus* in the Pyrenees (Angus 2010a) suggested that this species might have a more western distribution, a view strengthened by his subsequent discovery of this species widely distributed in the Pyrenees, in the apparent absence there of any other *Boreonectes* species.

In view of these discoveries the known distribution of *B. multilineatus* is reviewed and a detailed chromosomal comparison of all five West European *Boreonectes* is undertaken.

## Material and methods

The *B. multilineatus* used in this study is listed in Table 2. The sources of material of the other species are given in the primary references indicated in the captions to the illustrations of their karyotypes.

The methods used for preparing chromosomes and the handling of the data are those used by Dutton and Angus (2007) and subsequent papers on *Boreonectes*. The methods were described in detail by Angus et al. (2020).

**Table 2.** Localities of the *B. multilineatus* material used for chromosome analysis.

Country	Locality, date & collector	Coordinates	Number examined + reference
SWEDEN	Västerbotten, Åmsele, viii.1990, A.N. Nilsson	64.528°N, 19.350°E	3 ♂♂. Dutton and Angus 2007
SCOTLAND	Kirkcudbright, Clatteringshaws Loch, viii.1990, G. N. Foster	55.067°N, 4.282°W	4♂♂, 1♀. Dutton and Angus 2007
FRANCE	Lac d’Oncet, 11.ix.2010, F. Bameul.	42.927°N, 0.1335°E	2♂♂, 3♀♀. Angus 2010b
	Lac d’Anapéou, 30.vii.2011, F. Bameul.	42.926°N, 0.1284°W	5♂♂. Angus et al. 2015
	Etangs de Fontargente, 5.ix.2015, F. Bameul.	42.630°N, 1.710°E	1♂, 1♀. This paper
SPAIN	Ibón de las Isérias, 18.vii.2015, F. Bameul.	42.745°N, 0.479°W	3♂♂, 1♀. This paper
	Ibón de Anayet Este, 12.viii.2017, F. Bameul.	42.780°N, 0.4405°W	2♂♂. This paper
	Bielsa, “Ibón de Urdicito 1”, 29.viii.2015, F. Bameul.	42.669°N, 0.278°E	1♂, 2♀♀. This paper
	Chistén, “Ibón de Urdicito 2” Ibones de la Solana de Urdicito, 29.viii.2015, F. Bameul.	42.666°N, 0.286°E	
Northern IRELAND	Antrim, Garron Plateau above Glen Arrif, R. Anderson, 3.vi.2008	55.003°N, 6.062°W	4♀♀. Angus, 2008
IRELAND	Cork, NW Bonane, small lake 451 m a.s.l. on Glenlough Mountain K. Scheers, J. Bergsten & A. N. Nilsson, 10.vi.2018	51.746°N, 9.644°W	1 ♂, 2♀♀. This paper.

## Results

### *B. multilineatus*

**Figure 1.**
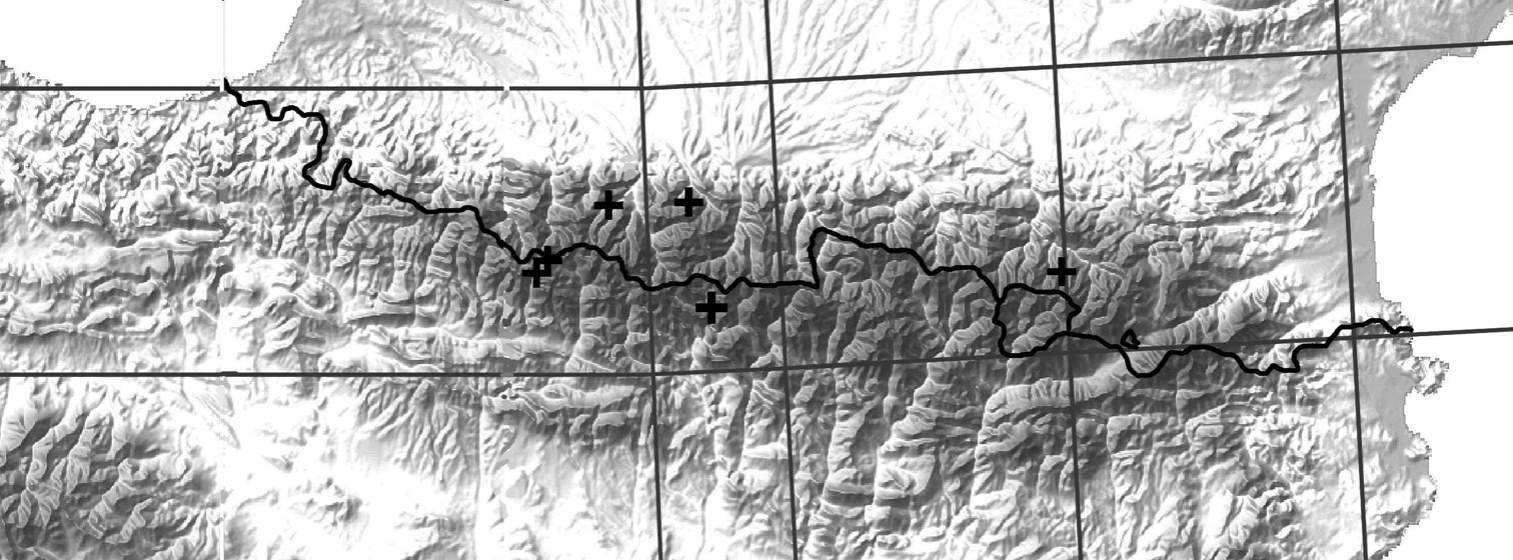
Map showing the Pyrenean localities for *B. multilineatus*. For details of the localities see Table 1. All specimens collected by Franck Bameul.

**Figure 2.**
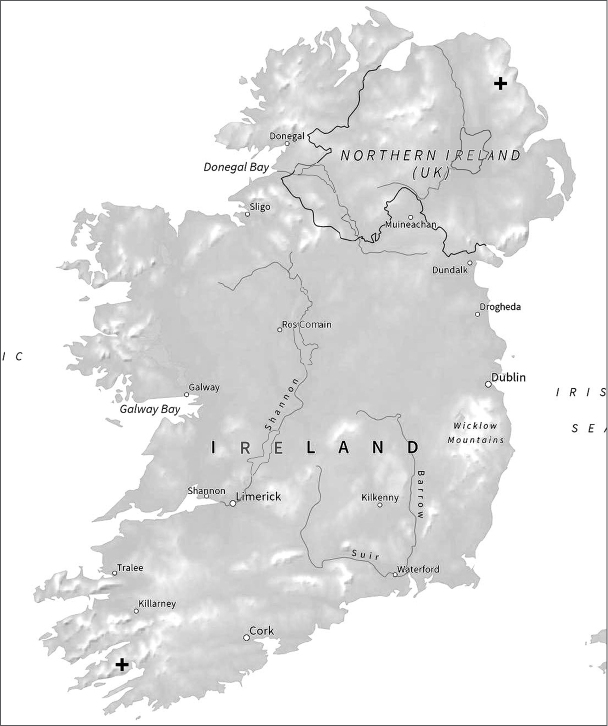
Map showing the Irish localities from which *B. multilineatus* chromosomes have been obtained.

**Figure 3.**
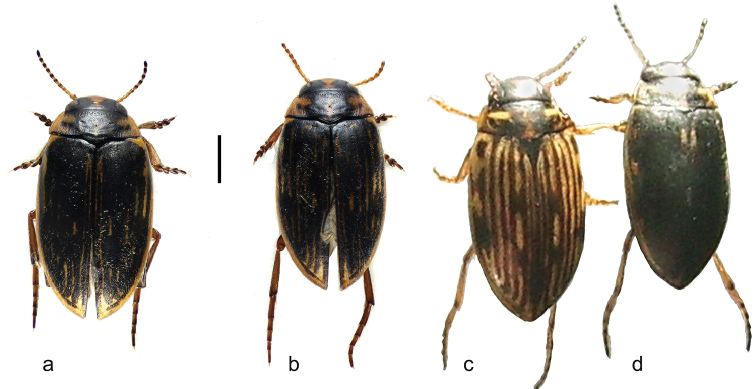
*
B. multilineatus* and *griseostriatus*, habitus **a, b***multilineatus*, Ireland, county Cork **a** ♀ **b** ♂ **c***B. griseostriatus*, Switzerland, Le Louché **d**B. griseostriatus var nigrescens Favre, Switzerland, Le Louché. Scale bar: 1 mm for (**a, b**); **c, d** are field photographs by Dr E. M. Angus.

The Pyrenean distribution of *B. multilineatus* is shown in Fig. 1. The species is widely distributed along the length of the Pyrenees, with localities in both France and Spain (see Table 2). All the specimens were collected by Franck Bameul. Fig. 2 shows the two Irish localities from which material yielding karyotypes has been obtained. Swedish and Scottish localities are listed in Table 2. British and Irish localities for *B. multilineatus* are given by Foster et al. (2016). The only other records for this species are from the Faroe Islands, the material in this case being identified by DNA analysis (Angus et al. 2017). Irish *B. multilineatus* are shown in Fig. 3a, b along with Swiss *B. griseostriatus* and *griseostriatus*var. nigrescens Favre. The Irish (Cork) *multilineatus* are much darker than the illustration given by Nilsson and Holmen (1995) as fig. 347, and are even darker than the *griseostriatus* shown as fig. 346. On the other hand, the Swiss *griseostriatus* shown in Fig. 3c is clearly paler, with well-separated dark lines. This specimen was taken along with the *griseostriatus*var. nigrescens shown in Fig. 3d.

**Figure 4.**
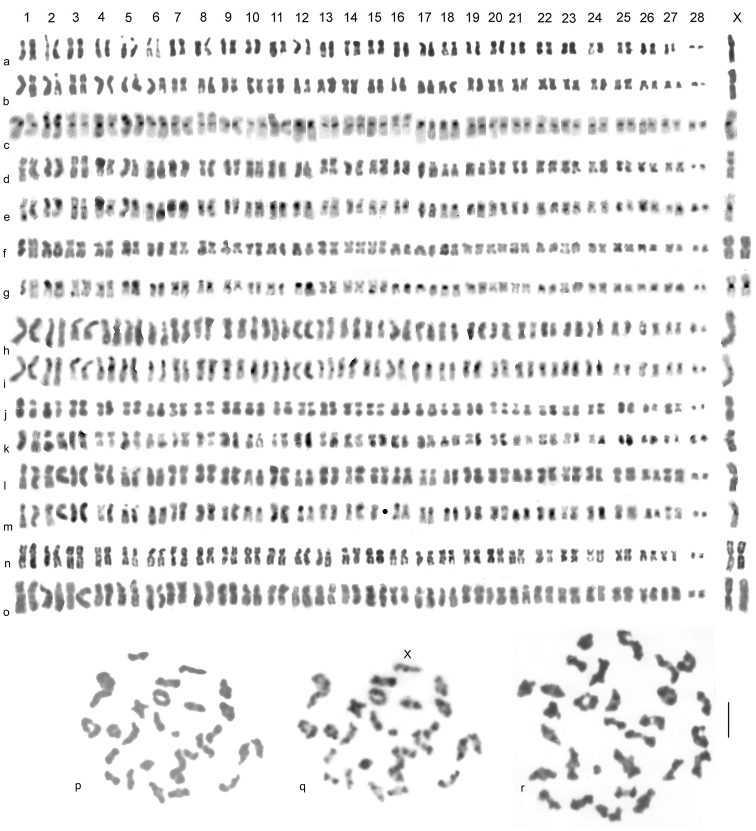
*
B. multilineatus* – mitotic chromosomes from midgut and testis (**a–o**), metaphase I of meiosis, from testis (**p–r**) **a** ♂, Sweden, Åmsele, plain (Giemsa stained), shown as fig. 2o by Dutton and Angus (2007)**b, c** ♂, Scotland, Clatteringshaws Loch **b** plain **c** C-banded **d–g** France, Hautes-Pyrénées, Lac d’Oncet **d, e** ♂ **f, g** ♀ **d, f** plain **e, g** C-banded, shown as fig. 3g–j by Angus (2010b)**h, i** ♂, France, Hautes-Pyrénées, Lac d’Anapéou **h** plain **i** C-banded, shown as fig. 5k, l by Angus et al. (2015)**j** ♂, France, Ariège, Etangs de Fontargente, plain **k** ♂, Spain, Ibon de Anayet Este **l, m** ♂, Spain, Ibon de Urdecito **l** plain **m** C-banded; One replicate of C-banded autosome 15 has been lost from **m**; **n** ♀, Ireland, Antrim, Garron Plateau above Glen Arrif **o** ♀, Ireland, Cork, NW Bonane **p, q** metaphase I, Spain, Provincia de Huesca, Ibones de Urdicito **o** plain **p** C-banded **r** Ireland, Cork, NW Bonane, Metaphase I, plain. Scale bar: 5 µm.

Mitotic chromosomes, arranged as karyotypes, are shown in Fig. 4a–n, and first metaphase of meiosis is shown in Fig. 4p–r. These cover all the localities from which *B. multilineatus* chromosomes have been obtained. The karyotype is made up of 28 pairs of autosomes and sex chromosomes which are X0 (♂) and XX (♀). The metric data are as follows: The longest autosomes measured 4.63 µ in the least condensed nucleus measured and 3.3 µ in the most condensed. The corresponding values for autosome 27 are 2.13 µ and 1.3 µ, and for autosome 28 (often dot-like) are 1.3 µ and 0.53 µ. The X chromosome, the longest in the nucleus, gave values of 7.2 and 5.5 µ. The Relative Chromosome Lengths (RCL, the length of each chromosome expressed as a percentage of the total haploid autosome length in the nucleus) range from 5.5 to about 2.5 in autosome 27 and 1.5 in autosome 28. The RCL of the X chromosome is about 6. Many of the autosomes have very similar RCLs (range about 3–3.8) so that adjacent pairs are often inseparable unless the centromere positions are clearly different (see Table 2). Note that measurement should be taken from simply Giemsa-stained chromosomes as C-banding often alters their apparent length. Five pairs of autosomes appear to have secondary constrictions. Detailed chromosomal comparisons are given in the next section.

### Chromosome comparisons

**Figure 5.**
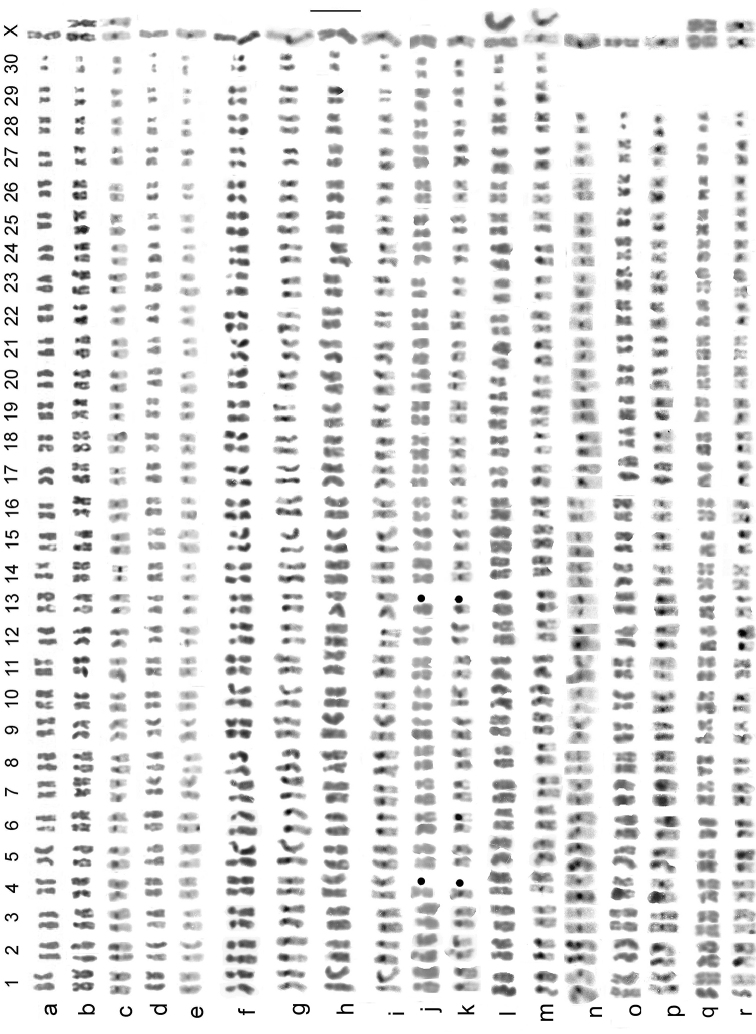
*
Boreonectes griseostriatus* (**a–m)** and *B. multilineatus* (**n–r**) at higher magnification, for detailed comparison. **a–e** northern localities **a** ♂, Sweden, Öregrund (shown as fig. 2a by Dutton and Angus 2007, and as fig. 2a by Angus 2008) **b, c** Finland, Sevettijärvi, ♀ **b** plain (Giemsa stained) **c** the same nucleus C-banded (shown as fig. 2b, c by Angus 2008) **d, e***B. g. strandi*, ♂, Norway, Bugøynes, **d** plain **e** the same nucleus C-banded (shown as fig. 2d, e by Angus 2008) **f–m** Alpine localities **f, g** ♂, Switzerland, Valais, pool by Le Louché **f** plain **g** the same nucleus, C-banded (shown as fig. 3d, e by Angus 2010) **h, i** ♂, Germany, Bavaria, Seeonalm **h** plain **i** the same nucleus C-banded (shown as fig. 3f, g by Angus 2010a) **j, k** ♂, France, Savoie, pool south of the Lac de Mont Cenis **j** plain **k** the same nucleus C-banded (shown as fig. 5c, d by Angus et al. 2015) **l, m** ♀, France, Savoie, Col du Petit Saint Bernard **l** plain **m** C-banded (shown as fig. 3c, d by Angus 2010a) **n–r***B. multilineatus*, details of the material given in Table 1**n** ♂, Scotland, C-banded **o, p** ♂, France, Lac d’Oncet **o** plain **p** C-banded **q, r** ♀, France, Lac d’Oncet **q** plain **r** the same nucleus, C-banded. Missing chromosomes indicated by solid circles. Scale bar: 5 µm.

**Figure 6.**
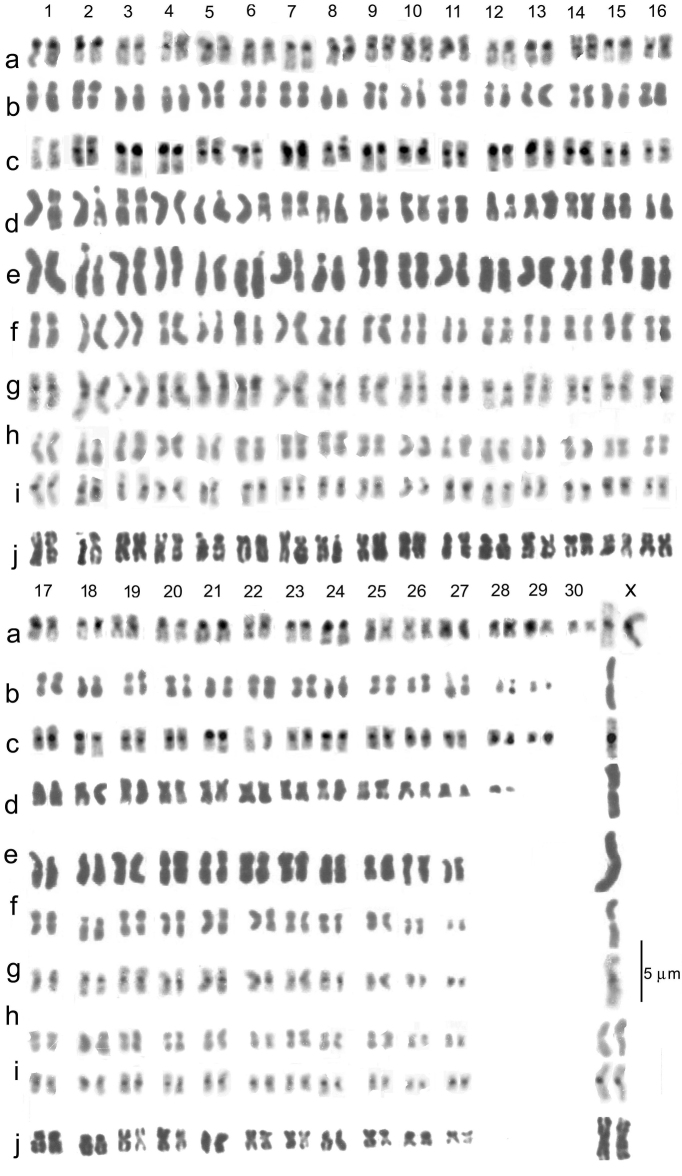
*
B. griseostriatus* (**a**), *B. inexpectatus* (**b, c**), *B. multilineatus* (**d**) and *B. alpestris* (**e–j**) at higher magnification, for detailed comparison **a***B. griseostriatus*, ♂, C-banded, Petit St Bernard (as fig. 2m) **c, d***B. inexpectatus*, paratypes **b** plain (Giemsa stained) **c** C-banded **c** is the nucleus shown as fig. 2i by Dutton and Angus (2007), here somewhat rearranged **d***B. multilineatus*, ♂, Scotland **e–j***B. alpestris***e** paratype ♂, Italy, Dolomites, Falcade, shown as fig. 2f by Dutton and Angus (2007)**f, g** ♂, Italy, Gran Paradiso, Colle del Nivolet **e** plain **f** C-banded, shown as fig.1b, c by Angus et al. (2017)**h, i** ♀, Switzerland, Ticino, San Bernardino pass **h** plain **i** C-banded, shown as fig. 1d, e by Angus et al. (2017)**j** paratype ♀, Switzerland, Ticino, Medeglia, plain, shown as fig. 2g by Dutton and Angus (2007). Scale bar: 5 µm.

**Figure 7.**
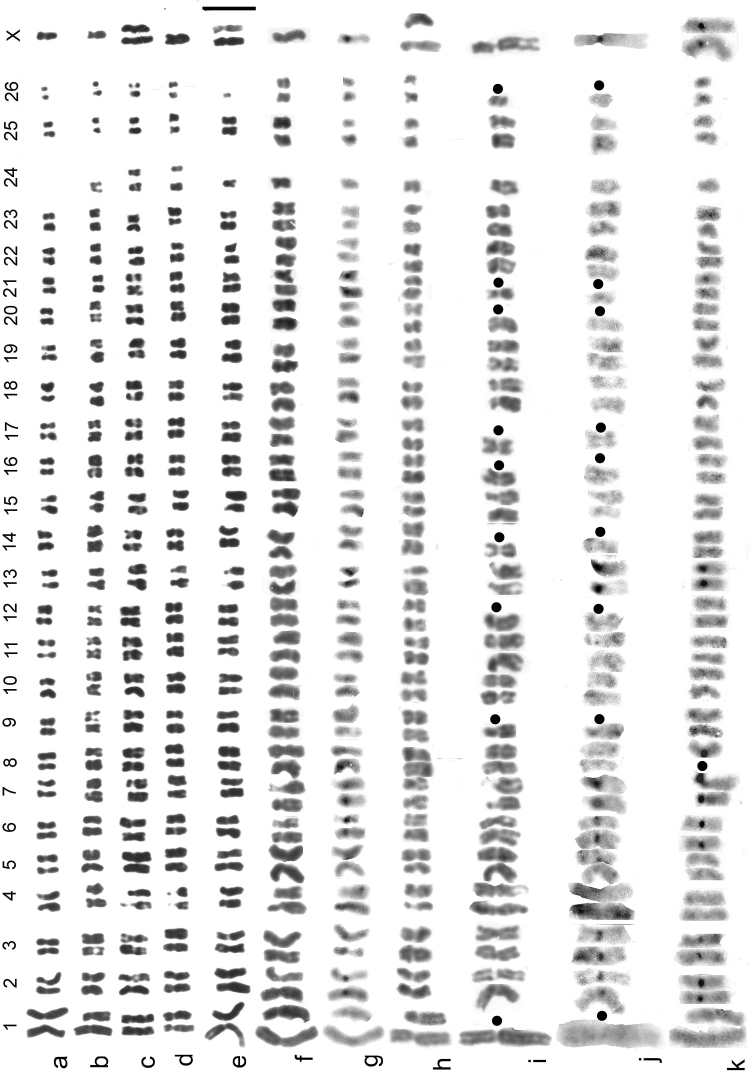
*
B. ibericus* at higher magnification, for detailed comparison **(a–k**) **a–c** paratypes, Spain, Peña Lara, plain (Giemsa stained) **a** ♂, autosomes 1 and 24 homozygous fused **b** ♂, autosomes 1 and 24 heterozygous for fusion **c** ♀, autosomes 1 and 24 homozygous unfused; shown as fig. 2j–l by Dutton and Angus (2007)**d, e** paratypes, Italy, Alpi Maritimi: plain **d** ♂, autosomes 1 and 24 homozygous unfused **e** ♀, autosomes 1 and 24 heterozygous for fusion, 1 replicate of autosome 26 lost (from Dutton’s unpublished MSc thesis) **f, g** ♂, Corsica, autosomes 1 and 24 heterozygous for fusion **f** plain **g** C-banded. Shown as fig. 3j, k by Angus (2010a) **h–j** Morocco, Moyen Atlas **h** ♀, plain, complete, autosomes 1 and 24 heterozygous for fusion **i, j** ♂, lacking 9 autosomes (positions indicated by solid circles), autosomes 1 and 24 heterozygous for fusion (unfused autosome 1 missing) **i** plain **j** C-banded. Shown as fig. 3o–q by Angus (2010b)**k** ♀ paratype, France, upper Maritime Alps (Hautes Alpes), Lac du Lauzet Superieur, C-banded, autosomes 1 and 24 heterozygous for fusion. Shown as fig. 2m by Dutton and Angus (2007). Scale bar: 5 µm.

Karyotypes for comparison of the West European species are shown in Figs 5–7. These are at a higher magnification than Fig. 4, so that it should not be necessary to zoom in on these if viewed as pdfs. Chromosome data are given in Table 3.

Centromere positions from Centromere Indices (CI), the length of the shorter chromosome as a percentage of the total length of the chromosome, based on Sumner (2003) and secondary constrictions (**2c**) of the *Boreonectes* species: m, metacentric, CI 46–50; sm, submetacentric, CI 26–45; sa, subacrocentric, CI 16–25. -C, no centromeric C-band. Note that secondary constrictions are identified by their appearing open in some preparations. No attempt has been made to stain for nucleolus organisers (NORs).

**Table 3.** Chromosome data.

Chromosome	* B. griseostriatus*	* B. multilineatus*	* B. alpestris*	* B. inexpectatus*	* B. ibericus*
1	sm	m	m - sm	-C sm	- C m (+ 24), sm (alone)
2	**2c**	**2c**	**2c**	m	m
3	sm - m	m - sm	m	sa	sm
4	m	m	m	sa	-C **2c**
5	**2c**	**2c**	**2c**	m	-C sm
6	sm	**2c**	sm	sm	m
7	sm - m	m	m	m	**2c**
8	**2c**	**2c**	sm	**2c**	-C m
9	m	m	m - sm	Sa	m (C very weak)
10	m	m	m	**2c**	-C m
11	m	**2c**	sm - sa	m	-C m
12	**2c**	sm - sa	sm - sa	sa	Sm
13	sa	m	m	sm	**2c**
14	m	m	m	sm	-C m
15	sm - sa	m	m	m	**2c** (C very weak)
16	m	**2c**	m	m	-C m
17	m	sa	sm	sm	-C m
18	m	sa	sm	m	-C sm
19	m	m - sm	m - sm	m	-C sm/sa
20	m - sm	m	m	m	-C sm
21	m - sm	m	m	sa	m (C sometimes weak)
22	m - sm	m	m	-C m	-C m
23	sm - sa	m	m	m	-C sm
24	sa	m	m - sm	sm	-C sa/sm
25	m	m	m	m	Sm
26	m	m	m - sm	m	-C sm
27	m	m	m	sm	–
28	sm (northern) m (Alpine)	m - sm (dot)	–	**2c**	–
29	sm (northern) m (Alpine)	–	–	m	–
30	sm (dot)	–	–	–	–
X	m	m	m	m	sm

#### *B. griseostriatus* and *B. multilineatus* (Fig. 5).

*B. griseostriatus* has the highest number of chromosomes of any of the species considered here, with 30 pairs of autosomes and X0/XX sex chromosomes. The metric data are: Measured lengths of autosome 1 2.53–1.99 µ, of autosome 29 1.73–0.87 µ, of autosome 30 (often dot-like) 1.28–0.71 µ. The values for the X chromosome are 4.42–2.3 µ. The RCLs of the autosomes range from about 4 (autosome 1) to about 2 (autosome 29) and about 1.6 (autosome 30). The RCL of the X chromosome is about 6.5. Other features of the chromosomes are given in Table 2. Four pairs of autosomes (2, 5, 8 and 12) have secondary constrictions (**2c**), but recognition of these requires preparations in which they are open, often more apparent on C-banded preparations as on autosome 2 in Fig. 5k, autosomes 5 and 8 in Fig. 5g. A secondary constriction on autosome 12 is suggested by Fig. 5b, c, d, e, these being Giemsa-stained and C-banded preparations from two specimens. There is a minor difference between northern (Fig. 5a–e) and Alpine populations (Fig. 5f–m) populations in that autosomes 28 and 29 are submetacentric in northern populations but metacentric in southern ones. All the chromosomes have distinct centromeric C-bands.

*B. multilineatus* has six clear secondary constrictions on autosomes 2, 5, 6, 8, 11 and 16, shown on the C-banded preparation in Fig. 5n and supported by the Giemsa-stained preparations shown in Fig. 4a, b. This is the highest number in this group. The constrictions on autosomes 2, 5, and 8 appear to match those of *B. griseostriatus*, but the one on autosome 11 is unmatched in the *B. griseostriatus* karyotype, though one is present on pair 12. The secondary constriction on autosome 16 of *B. multilineatus* appears completely unmatched in *B. griseostriatus*. The metric data are given in the preceding section. All the chromosomes have distinct centromeric C-bands.

#### *B. alpestris* and *B. multilineatus* (Fig. 6)

*B. alpestris* has a karyotype at first sight closely resembling that of *B. multilineatus* but has only 27 pairs of autosomes plus the usual X0 or XX sex chromosomes. The metric data are: measured lengths of autosome 1 3.75–2.68 µ, of autosome 26 2.25–2.02 µ, of autosome 27 1.97–1.66 µ and of the X chromosome 6.25–3.3 µ. The RCLs range from about 4 (autosome 1) to about 2.15 (autosome 26) and about 1.8 (autosome 27), while that of the X chromosome ranges from about 8.35–5.79. Other features are given in Table 2. All the chromosomes have clear centromeric C-bands, and only 2 secondary constrictions, on autosomes 2 and 5, are apparent. The decrease in RCL between the 2 smallest autosomes (pairs 26 and 27) is clearly less than between those of *B. multilineatus* (pairs 27 and 28).

#### *B. inexpectatus* and *B. griseostriatus* (Fig. 6)

*B. inexpectatus* has a karyotype comprising 29 pairs of autosomes and X0, XX sex chromosomes, and thus, in terms of numbers of chromosomes, is the species coming closest to *B. griseostriatus*. The metric data are: measured lengths of autosome 1 2.8–2.4 µ, of autosome 28 1.16–1.06 µ, of autosome 29 0.75–0.63 µ and of the X chromosome 4.16–3.12 µ. The RCLs range from about 5.9 (autosome 1) to about 2.25 (autosome 28) and about 1.4 (autosome 29), while that of the X chromosome is about 7. Other features are given in Table 2. Autosomes 1 and 22 lack centromeric C-bands and autosomes 8, 10 and 28 have secondary constrictions. Autosome pairs 3, 4, 9, 12, 21 and 24 have particularly heavy subacrocentric C-bands, and autosome pair 7 has equally heavy submetacentric ones. The *B. inexpectatus* karyotype appears unlike that of any other species. To date this species is known from only one locality, the Lac de Lauzet inférieur, the smaller and higher of the two Lauzet lakes. Dutton and Angus (2007) mention chromosome preparations from three males, done on November 13^th^ 1998, and a further male and two females done on November 30^th^ that year. The 13^th^ November preparations gave karyotypes agreeing with each other, but the preparations done on the 30^th^ failed – nothing was photographed. No DNA data are available.

#### *B. ibericus* (Fig. 7)

*B. ibericus* has the most distinctive karyotype of any of the West European species. It has 24–26 pairs of autosomes, with a fusion-fission polymorphism involving pairs 1 and 24. The sex chromosomes are X0 (♂), XX (♀), with the X chromosome clearly submetacentric, as against metacentric in all the other species. 15 pairs of autosomes lack centromeric C-bands, 1 pair (No. 8) may be with or without C-bands, and 3 pairs have them very weak. The metric data are: measured lengths of autosome 1 5.62–2.8 µ (fused with autosome 24), 3.93–2.5 µ (unfused), of autosome 24 1.25–1 µ, of autosome 26 1.45–0.62 µ and of the X chromosome 6.66–3.12 µ. Secondary constrictions are present on autosome pairs 4, 7, 13 and 15.

## Discussion

The West European species of *Boreonectes* show a striking diversity of karyotypes, in sharp contrast to their genetic (DNA) differences which are very slight (Angus et al. 2017). The two studied southeast European species (*B. macedonicus* (Georgiev, 1959) and *B. riberae* (Dutton et Angus, 2007)) are slightly more distant genetically and the Tibetan *B. emmerichi* (Falkenström, 1936) much more so, in fact closer to American “*griseostriatus*”, although its karyotype is very similar to that of *B. macedonicus* (Angus et al. 2015). This suggests that in western Europe chromosome diversification has been a driver of speciation. Five species are involved, of which two, *B. griseostriatus* and *B. multilineatus*, have distributions extending into northern Europe. The extent of the northern distribution of *B. griseostriatus* requires clarification, as does the possibility of its existence in the Nearctic. It is very easy to regard *B. griseostriatus* as a primarily northern species with “glacial relict” populations in the Alps. However, as Ignacio Ribera has stressed in conversations, much if not all of these northern parts of its range would have been covered by ice sheets over much of the Last Glaciation, and hence uninhabitable, so the southern populations are likely to be the older ones. In the case of *B. multilineatus*, the eastern extent of its northern range requires clarification and it seems possible that Pyrenees may be the oldest part of its range. Nilsson and Holmen (1995) record *B. griseostriatus* only from coastal parts of Finnmark (Norway), with the inland parts occupied by *B. multilineatus*. This inland Finnmark material should be checked in view of the stripy *B. griseostriatus* taken at Sevettijärvi near Inari in Finnish Lapland. Fortunately, the two species are separable by details of the male genitalia – aedeagus and parameres.

**Figure 8.**
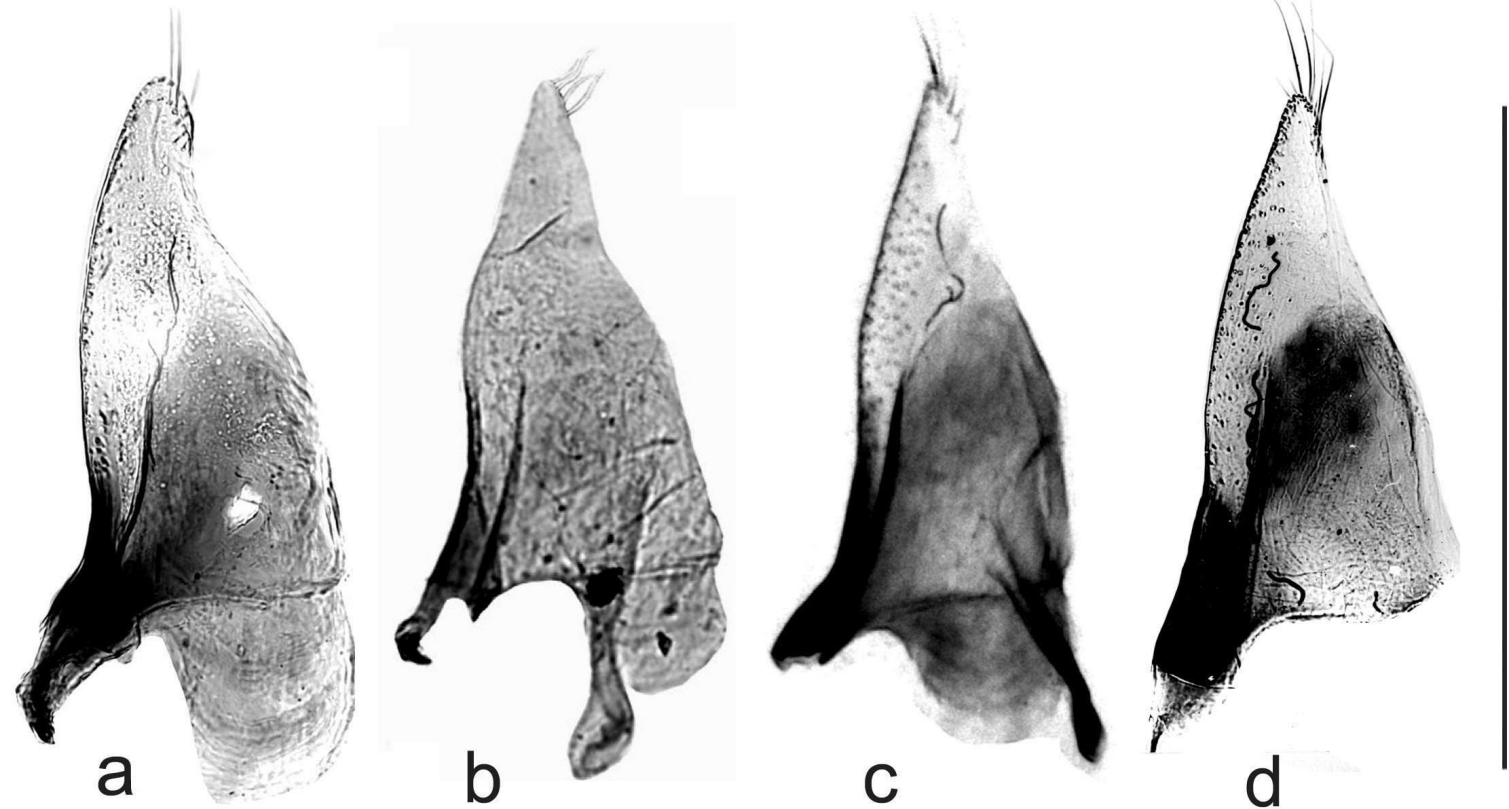
*
Boreonectes* parameres. **a***B. multilineatus*, Ireland, county Cork **b–d***B. griseostriatus***b** Starunia fossil **c** Norway, Tjøme **d** Sweden, Öregrund. The apodeme on the inner face is present in **b** and **c** but lost from **a** and **d**; **d** also lacks the basal lobe. Scale bar: 0.5 mm.

There is some fossil evidence for these beetles in central Europe during the Last Glaciation. *Boreonectes* is among the beetles at the famous Woolly Rhinoceros site at Starunia near Lvov in the western Ukraine (Angus 2010a). The material includes a male from which the aedeagus has been dissected. The median lobe and one paramere are present and the paramere is shown, along with those of modern *B. griseostriatus* and *B. multilineatus*, in Fig. 8. The apical parts of these parameres are very prone to shrivelling when dried, and the Irish *B. multilineatus* (Fig. 8a) was transferred to DMHF immediately after dissection and hence shows the true shape of the apex. The two *B. griseostriatus* (Fig. 8c, d) were also treated this way and so show their true shape. The fossil (Fig. 8b) was laterally compressed but has preserved its shape (as often happens with Pleistocene fossils), and very clearly matches *B. griseostriatus* rather than *B. multilineatus*. The elytra are pale with well-separated dark stripes. The bleaching of the darker more sclerotised main part of the parameres is an artefact of its having been preserved in oil (and salt) and is typical of many of the Starunia fossils. The *Helophorus* Fabricius, 1775 of the Starunia site were investigated by Angus (1973), who listed 9 species, with a mixture of East Siberian and European species. Angus (1982), on the basis of their karyotypes, separated *H. aquaticus* (Linnaeus, 1758) and *H. aequalis* Thomson, 1868, both European species, and showed that both were present among the Starunia fossils. Angus (1998) showed that the two Starunia fossils regarded in the 1973 account as being *H. glacialis* (Villa et Villa, 1833), were in fact *H. griseus* (Herbst, 1793) and showed how the aedeagi of that European species could be distinguished from Tibetan *H. montanus* d’Orchymont, 1926. This brings the total number of species to 10. Angus gave the age of the Starunia fauna as about 23,000 years, based on radiocarbon dating of collagen from the Woolly Rhinoceros done in the Smithsonian Institution in Washington DC (SI-642), which would date it is shortly before the maximum extension of the ice sheets of the Last Glaciation. However, subsequent investigations have resulted in a somewhat older date of 33,000–40,000 years (Kuc et al. 2012), which puts it squarely in the Cold-Continental phase which followed on from the somewhat warmer Upton Warren Interstadial. Faunas of this period, in England, can be quite rich and include a mixture of European and East Palaearctic species. Coope (1977) gives an account of temperature fluctuations during the Last (Devensian) Glaciation. The Last (Eemian or Ipswichian) Interglacial dates to about 120,000 years ago and was succeeded by a cold treeless episode. Then, at about 60,000 years ago there was an episode, the Chelford Interstadial, in which Northern Coniferous forest developed in England. This was followed by a return to tundra conditions, which lasted till about 43,000 years ago and was followed by the Upton Warren Interstadial, an episode with warm but treeless conditions and a predominantly European fauna (Girling 1974; Coope and Angus 1975). After the thermal maximum of the Upton Warren Interstadial there was a gradual change to a colder more continental climate with faunas including often abundant Siberian and Tibetan species. This colder phase lasted from about 40,000 to about 25,000 years ago when temperatures lowered still further as the maximum extent of the ice sheets approached. The oldest known of these post thermal maximum faunas was at Queensford gravel pit near Dorchester on Thames and was dated to 39,300 +/- 1350 years ago (Coope 1985). This was a rich fauna apparently dating from the oldest age given for the Starunia site. *Boreonectes* sp. was among the included species. These faunas give an indication of the full-glacial environments of Central Europe in which *Boreonectes griseostriatus* lived. Unfortunately, there are no identifiable fossils of *B. multilineatus*. There are *Boreonectes* fossils in England, but these are isolated elytra which, although clearly marked with dark stripes on a pale background, could belong to either *B. griseostriatus* or *multilineatus*.
